# Evolution of crystal structures in GeTe during phase transition

**DOI:** 10.1038/s41598-017-01154-z

**Published:** 2017-04-19

**Authors:** Kwangsik Jeong, Seungjong Park, Dambi Park, Min Ahn, Jeonghwa Han, Wonjun Yang, Hong-Sik Jeong, Mann-Ho Cho

**Affiliations:** 1 Institute of Physics and Applied Physics, Yonsei University, Seoul, 120-749 Republic of Korea; 2 School of Integrated Technology, Yonsei University, Incheon, 406-840 Republic of Korea

## Abstract

We investigated changes in the crystal structure of GeTe during its phase transition. Using density functional theory (DFT) calculations, four possible crystal structures were identified: R3m, P1, Cm, and Fm3m. Among these, P1 and Cm were examined here for the first time. By calculating the internal energy of the crystal volume change, we verified that P1, R3m, and Cm can coexist in crystalline GeTe. The X-ray diffraction spectra of annealed and laser-irradiated GeTe films revealed coexisting P1 or R3m and Cm. In addition, we confirmed that Cm transforms into P1 or R3m after laser irradiation. The presence of these new structures was revealed in the crystal Raman spectra. Many of the Raman peaks in the crystalized GeTe could be explained by the coexistence of various structures. By calculating the band gaps of these structures, we also found that a structural transformation induces a change in the crystal resistance, owing to differences in the band gaps of individual structures. The generation of new crystal structures suggests a facile phase change and instability during the structural transformation.

## Introduction

Germanium telluride (GeTe) has been extensively studied for many years owing to it alluring phase transition characteristics^1^. The transformation of GeTe from the amorphous phase to the crystal phase induces a significant change in its resistance, which can be advantageous for devices such as phase change random access memory (PRAM)^2^. Therefore, this phase transition of GeTe has been of significant interest. Although the characteristics of amorphous and crystalline GeTe have been steadily and extensively investigated, including their bond length, bonding properties, and electronic structures^3, 4^, several properties remain elusive, even for crystalline GeTe that is simpler than amorphous GeTe. For example, although it is well known that GeTe has a typical crystal structure of R3m in atmospheric ambient conditions^5^, various band gaps of GeTe, in the 0.3–0.8 eV range, were reported^4, 6, 7^. This variation in measured band gaps can be satisfactorily explained for amorphous GeTe. Jean *et al*. reported that the optical band gap of amorphous GeTe can vary from 0.1 eV to 0.6 eV with a fraction of tetrahedral Ge atoms^8^. On the other hand, although the reported band gap for crystalized GeTe varied in the 0.3–0.8 eV range^4, 6, 7^, the variation cannot be clearly understood based on the fraction of Ge atoms. Characteristic band gap values of 0.3 eV and 0.8 eV can be explained by crystallized GeTe structures for Fm3m and R3m, respectively^9^. However, moderate band gap values for crystalline GeTe cannot be explained in terms of R3m and Fm3m structures only. In addition, because R3m is stable in atmospheric ambient conditions, possible Raman peaks can be well defined using the group theory formalism. However, even in well-crystallized GeTe, unexpected Raman peaks have been reported in many previous studies^10–14^. Moreover, the shape of the Raman spectra of crystalized GeTe depends on the synthesis conditions. These results strongly suggest that additional crystalline structures can be present that have not been reported yet.

In this study, to establish a relationship between band gaps and crystalline structures in GeTe, we investigated its crystal structure in detail, using a combination of experimental and computational methods. The sample structure was modified by thermal annealing and pulsed laser irradiation, and this modification was confirmed by performing the Raman and X-ray diffraction (XRD) spectral analyses. In addition, using density functional theory calculations, we observed several new structures with locally minimal energy. By comparing the structural and bonding characteristics determined for the samples using the XRD and Raman analyses with those obtained from DFT calculations, we confirmed the generation of new structures. The crystalline structure predicted by our calculations was consistent with that determined experimentally. In particular, we observed a crystalline structure with intermediate bonding characteristics, indicating that the relaxation of the Peierls distortion plays the main role in the phase transition process and the formation of intermediate bonds. Moreover, analysis of chemical ordering characteristics showed that the phase of GeTe can be quite easily changed.

## Methods

### Sample preparation and measurements

An 80-nm-thick GeTe film was deposited at room temperature by ion beam sputtering deposition (IBSD) using one GeTe target. We used thermally grown 300-nm-thick SiO_2_ on Si(100) as a substrate. A 25-nm-thick SiO_2_ layer was subsequently deposited onto the GeTe film by IBSD, without breaking the vacuum condition. To crystalize a GeTe sample, annealing of GeTe by a rapid thermal process (RTP) was conducted for 15 min at 250 °C under N_2_ ambient^15^. To modify the crystal structure, laser irradiation in the direction normal to the sample was performed using a KrF pulsed laser (wavelength = 248 nm; duration = 25 ns). During the irradiation process, the chamber for irradiation was maintained under a vacuum of 1 × 10^−7^ Torr. The energy density of the laser was chosen to trigger a resistance change, which served as the evidence of the phase transition. The change in resistance was confirmed in the sample on a 1 cm × 1 cm area, using the 2-point contact method. To prevent a phase change induced by the electrical Joule heating, the resistance was measured for a low DC voltage (0.05 V). By laser irradiation, we obtained three types of samples: 1) a crystallized sample (CS) with the resistance of ~50 Ω, prepared by annealing only; 2) a partially reset sample (PRS) with the resistance of ~430 Ω, prepared by a weak laser pulse irradiation (35 mJ/cm^2^); and 3) a more partially reset sample (LRS) with the resistance of ~17 kΩ, prepared by a higher-power laser pulse irradiation (45 mJ/cm^2^). To analyze the crystal structures of these samples, we used 2-theta XRD with Cu K_a_ X-rays, and compared the results to the diffraction intensities calculated using Rietveld refinements with the FullProf program^16^. Microstructural changes in the crystal samples were also verified using the Raman spectroscopy that was conducted using the backscattering geometry. An ND:Yag laser serving as a low-power (2 mW) illumination source was used for eliminating thermal effects.

## Calculations

**Figure 1:**
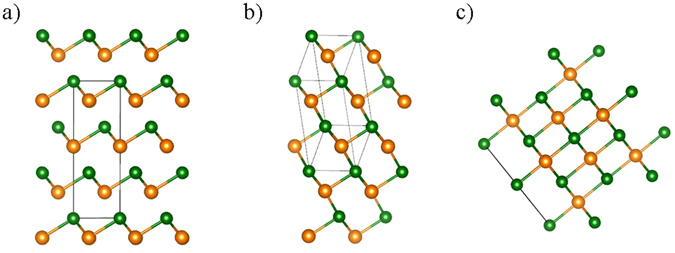
Atomic images of (**a**) P1 or R3m, (**b**) Cm, and (**c**) Fm3m structures. The P1 and R3m structures feature three short and three long bonds. P1 and R3m cannot be distinguished from structural images owing to their structural similarity. The Cm structure features two short, two intermediate, and two long bonds. The Fm3m structure features six identical bonds.

To investigate the changes in the crystal structure of GeTe, we performed DFT using the Vienna ab-initio simulation package (VASP)^17^. First, we optimized the geometry of R3m and the face centered crystal (Fm3m) structure. For all calculations, we used the GGA-PBE function with the DFT-D2 van der Waals correction^18, 19^. To make the k-spacing less than 0.25/Å, we chose a 9 × 9 × 9 grid of k-points. The cut-off energy was set to 500 eV. Geometrical optimization was performed using the RMM-DIIS algorithm, iterating until the 0.01 eV/Å condition was satisfied. All calculation conditions were determined based on the differences between internal energies of different structures; this difference was under 0.1 meV/unit cell. In the primitive cell, the geometry-optimized fractional atom positions were (0, 0, 0), (0.52, 0.52,0.52) for the R3m structure and (0, 0, 0), (0.5, 0.5, 0.5) for the Fm3m structure. We induced the symmetry breaks of x = y ≠ z and x ≠ y ≠ z in these structures. For the structure with x ≠ y ≠ z, we chose the atomic positions of the initial structure to be (0, 0, 0) and (0.53, 0.52, 0.51). For the structure with x = y ≠ z, we chose the atomic positions of the initial structure to be (0, 0, 0) and (0.53, 0.50, 0.50). Initial lattice parameters remained the same as the lattice parameters of the R3m structure. Using the optimization, we found that these symmetry-broken structures were optimized in the new phase. The P1 structure was characterized by the x ≠ y ≠ z symmetry condition while the Cm structure was characterized by the x ≠ y = z symmetry condition. Small perturbations of atomic positions, ensuring the same symmetry scheme, did not affect the final optimized structures. The calculated lattice parameters and atomic positions are summarized in Table 1 and compared with reported reference values and the results of our XRD analysis. For the R3m structure, the difference between the reported experimental lattice parameter and our calculation was under 0.5%. Therefore, our DFT calculations satisfactorily describe real GeTe. To confirm the stability of the crystal structures P1, R3m, Cm, and Fm3m under volume changes, we applied a hydrostatic pressure to our structures. Under the applied hydrostatic pressure, we geometrically optimized each structure, and then verified the volume and energy changes. In addition, to confirm that these new structures reflect the microstructure of real crystals, we calculated their phonon properties, including the Raman spectra. In these phonon calculations, a 2 × 2 × 2 supercell was used, the cutoff energy was 500 eV, and the grid had 5 × 5 × 5 k-points. We used the GGA-PBE function with the DFT-D2 van der Waals correction. For thermodynamic analysis, the Gibbs free energy was extracted from the phonon calculation results. Finally, to predict the electronic structures of individual new crystal structures, band gap calculations were performed. To predict the exact band gap, band structures were calculated using the HSE06 hybrid functional^20^. The calculated structures are shown in Fig. 1.

**Table 1:** Geometric characteristics of GeTe crystal structures.

	R3m					P1	Cm		Fm3m	
	Lattice									
	Cal	our XRD			Ref^21^	Cal	Cal	our XRD	Cal	Ref*^32^
		50 ohm	430 ohm	17k ohm						
**a**	4.260	4.236	4.241	4.267	4.281	4.263	4.287	4.287	4.178	4.260
**b**	4.260	4.236	4.241	4.267	4.281	4.261	4.201	4.201	4.178	4.260
**c**	4.260	4.236	4.241	4.267	4.281	4.262	4.201	4.201	4.178	4.260
	**Cell Angle**									
	**Cal**	**our XRD**			**Ref**	**Cal**	**Cal**	**our XRD**	**Cal**	**Ref**
		**50 ohm**	**430 ohm**	**17k ohm**						
**α**	58.501	58.970	58.54	58.62	58.36	58.47	59.14	59.14	60.00	60
**β**	58.501	58.970	58.54	58.62	58.36	58.47	59.30	59.30	60.00	60
**γ**	58.501	58.970	58.54	58.62	58.36	58.47	59.30	59.30	60.00	60
	**Atom potion**									
	**Cal**	**our XRD**			**Ref**	**Cal**	**Cal**	**our XRD**	**Cal**	**Ref**
		**50 ohm**	**430 ohm**	**17k ohm**						
**Ge x**	0.000	0.000	0.000	0.000	0.000	0.000	0.000	0.000	0	0
**Ge y**	0.000	0.000	0.000	0.000	0.000	0.000	0.000	0.000	0	0
**Ge z**	0.000	0.000	0.000	0.000	0.000	0.990	0.000	0.000	0	0
**Te x**	0.523	0.523	0.523	0.523	0.522	0.528	0.559	0.559	0.5	0.5
**Te y**	0.523	0.523	0.523	0.523	0.522	0.523	0.501	0.501	0.5	0.5
**Te z**	0.523	0.523	0.523	0.523	0.522	0.513	0.501	0.501	0.5	0.5

*Measured in 773 K.

## Results and Discussion

**Figure 2:**
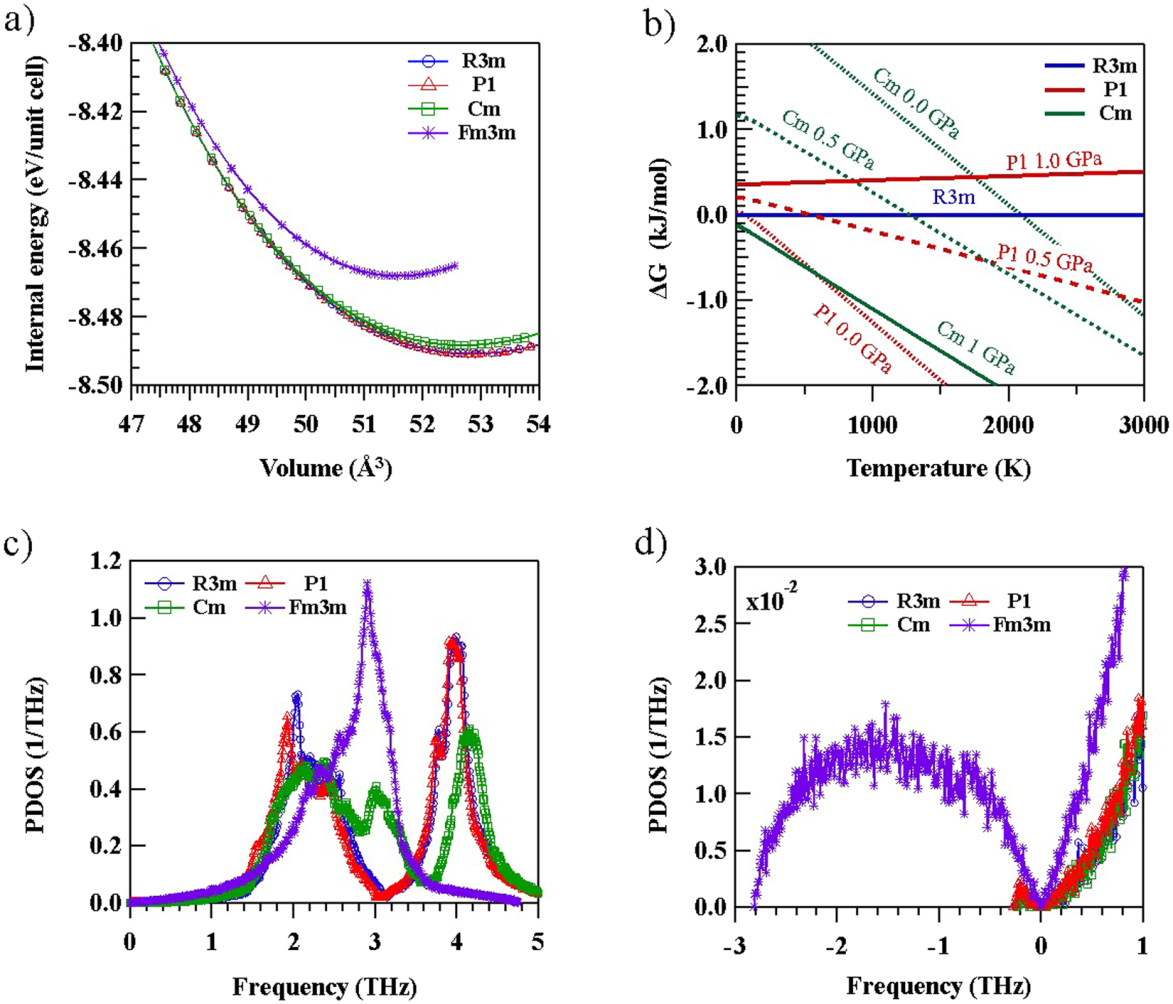
(**a**) Calculated volume versus internal energy for the various studied crystal structures. P1 and R3m exhibit stable characteristics under ambient pressure, whereas Cm becomes stable under compression. (**b**) Calculated difference between the Gibbs free energies of different structures. The Gibbs free energy of R3m serves as a reference. (**c**) Calculated phonon density of states (PDOS) for P1, R3m, and Cm. (**d**) Amplified view of the of negative frequency region. Only Fm3m exhibits PDOS at negative frequencies, implying structural unitability.

To confirm the structural stability of GeTe under the volume change, we investigated the relationship between volume and internal energy for some modified structures of GeTe. In atmospheric ambient conditions under zero stress, the volumes of the modified GeTe structures were 52.78, 52.82, 52.59, and 51.59 Å^3^ for R3m, P1, Cm, and Fm3m, respectively (Fig. 2a). Among these structures, P1 and R3m were the most stable. Since the energy difference between the P1 and R3m structures was under 0.1 meV/unit cell (which is within the calculation error range), P1 and R3m structures can coexist. On the other hand, the Cm structure has a slightly higher internal energy (25.5 meV/unit cell) than P1 and R3m. In the presence of compressive stress, which can be induced in GeTe during the amorphous-to-crystalline phase transition, the energy difference becomes smaller. In this case, the internal energy difference between Cm and P1 or R3m decreases. Thus, an external stress changes the internal energy of GeTe sufficiently to promote the generation of Cm; consequently, Cm structures can be induced in real devices during crystallization^21^. On the other hand, it is difficult to form Fm3m structures owing to a high internal energy difference among these structures. In particular, under higher compressive stress, the internal energies of P1, R3m, and Cm approach the internal energy of Fm3m. Our calculations are consistent with many previous reports of GeTe transformation into Fm3m under very high compressive stresses^9, 22, 23^. Without any external pressure applied on GeTe, Ge–Te bonds split into shorter bonds and longer bonds. As the pressure increases, the difference between the two types of bonds gradually decreases; finally, the Ge–Te bond lengths merge into narrow distributions for the first nearest neighbors. In this case, GeTe can be still considered as a distorted rocksalt structure with Ge and Te uniquely located on fcc sublattices. Therefore, owing to the instability of the Fm3m structure, these structures form only in high-pressure conditions that shorten the long van der Waals bond, resulting in the situation in which the six bonds become nearly identical.

Since the phonon density of states (PDOS) reflects internal energy differences attributed to different structures, we investigated in detail the changes in the PDOS associated with the structural changes in GeTe. The PDOS of the P1 structure was similar to that of the R3m structure, except for some shifting of the first peak (Fig. 2c). In the PDOS of the Cm structure, a new peak was observed between the two peaks at 2.1 THz and 3.9 THz that correspond to the main peaks in R3m (P1), indicating that the microstructure of Cm differs from those of R3m and P1. On the other hand, the PDOS of Fm3m was not similar to those of the other structures. In particular, some PDOS in the negative frequency range was observed for Fm3m (Fig. 2d). Because the PDS at negative phonon frequencies means that the structure can change to a stable system with distortion, it also indicates that a transformation of the structure into another structure can be preferred^24^. Therefore, such a negative-frequency PDOS can serve as an index of structural instability. Except Fm3m, no other structures had PDOS in the negative frequency range. Thus, we predict that P1, R3m, and Cm structures are stable in ambient conditions, whereas Fm3m is not stable under ambient pressure. The results show that rocksalt GeTe without bond splitting is not stable, because the Peierls distortion can release the repulsive energy and stabilize the structure. The changes in the internal energy and phonon density states strongly suggest that new structures, such as P1 and Cm, can exist under the film growth and operation conditions used for PRAM.

To determine the possibility of phase transitions for various structures, we performed thermodynamic analysis with DFT calculations. By performing the phonon calculations for several compressive stress conditions, we obtained the Gibbs free energy values for different structures, and compared the results with the Gibbs free energy of R3m as a reference; this comparison is shown in Fig. 2b. Based on the thermodynamic analysis, we concluded that the phase transition to Fm3m can occur in the presence of a very high pressure, while the formation of Fm3m in ambient conditions is not possible. The Gibbs free energy difference between Fm3m and R3m was larger than 10 kJ/mol for compressive pressures up to 1 GPa. Without compressive stress, R3m is the most favored structure for temperatures in the 0–150 K range. For temperatures above 60 K, the Gibbs free energy difference between P1 and R3m becomes negative, indicating a favored formation of P1. It is reasonable that P1 is formed as distorted R3m at high temperatures. The difference between the Gibbs free energy of Cm and that of R3m becomes negative at temperatures above 2100 K. In addition, since the Gibbs free energy of P1 is always lower than that of Cm, Cm structures cannot be generated without pressure. With compressive pressure, the difference between the Gibbs free energies of the different phases is changed. In particular, the Gibbs free energy of P1 increases, while that of Cm decreases. As a result, under the compressive stress of 0.5 GPa, the transformation to Cm is most favored at temperatures above 1850 K, while that to R3m is most favored at temperatures below 600 K. Increasing the stress to 1.0 GPa favors the transformation to Cm in all regions, i.e., the difference between the Gibbs energies of R3m and P1 is always positive. Because a small amount of stress can modulate the favored phase, P1, R3m, and Cm structures can coexist in films, and the extent to which these structures are present in each phase is determined by pressure and temperature conditions. Within PRAM operation temperatures (300 K to 1000 K), as compressive stress can be increased up to 1 GPa, phase transitions can occur reversibly, P1-R3m-Cm.

**Figure 3:**
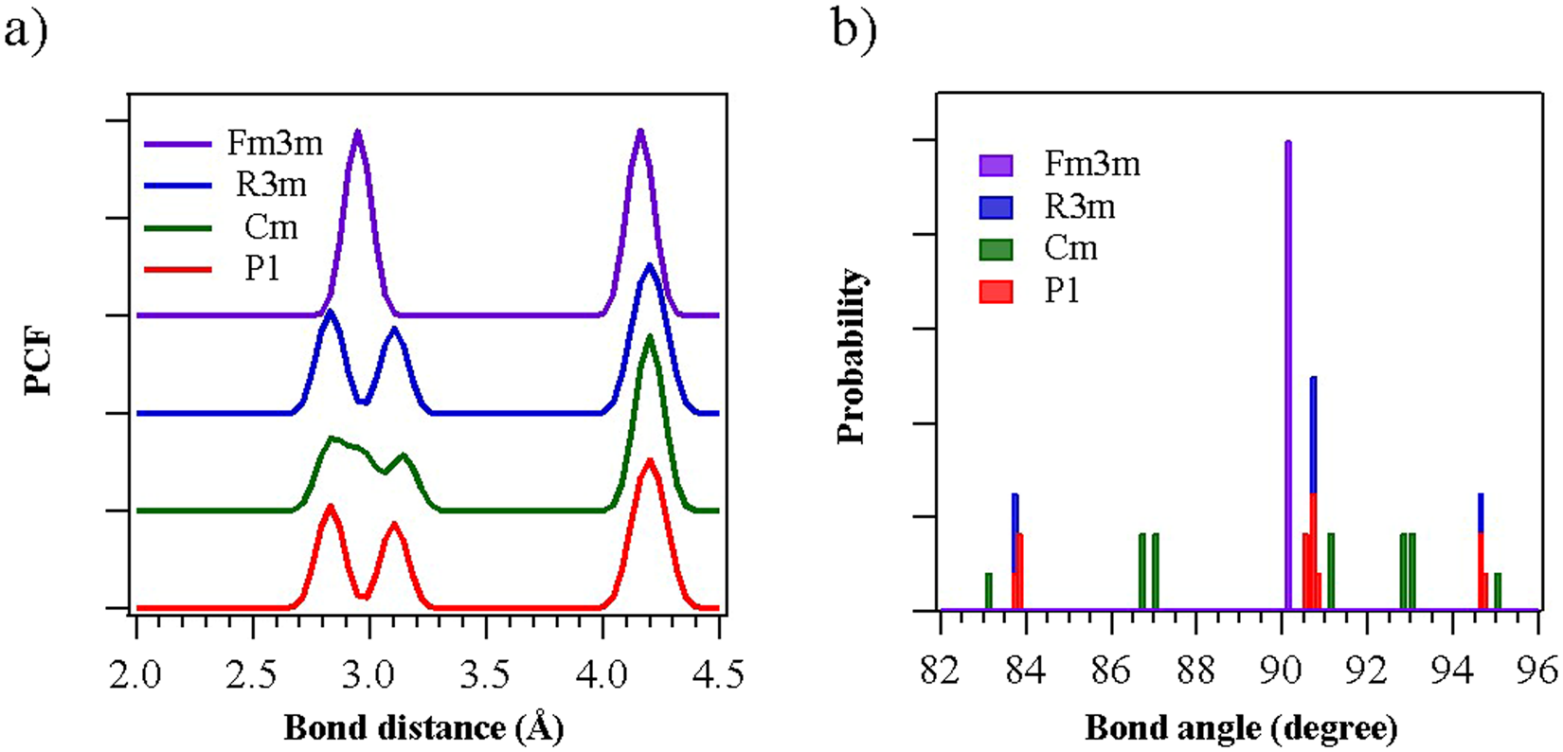
(**a**) Calculated pair correction functions and (**b**) angle distribution, for each of the possible GeTe structures. The geometries of P1 and R3m are very similar, while that of Cm is different.

Lattice parameters and atomic positions of different structures are listed in Table 1. As shown in Table 1, the lattice parameters are very similar to the reference values and the lattice constants a, b, and c are nearly the same, except for Cm. In particular, the lattice constant of a is not the same as those of b and c for Cm: a ≠ b = c. Similarly, the cell angles are the same for all structures, except for Cm, for which α ≠ β = γ. To investigate the structural differences between similar structures, pair correlation functions (PCFs) of bond distance and angle distribution were calculated, and are shown in Fig. 3. Although the bond length of P1 was very similar to that of R3m, P1 exhibited some angular distortion compared with R3m (Fig. 3a). Such an angular distortion can contribute to the phase transition of GeTe: by means of this small distortion, GeTe can be transformed into another structure without any significant change in the energy. Therefore, both P1 and R3m structures can coexist under the same conditions; in addition, transformations between P1 and R3m can occur easily at room temperature. Owing to the unique properties of the small distortion distinguishing these two structures and the easy transformation between them, it is difficult to experimentally discriminate between the two structures in GeTe films. Therefore, P1 and R3m structures have been generally considered as the same structure, despite their symmetry differences. On the other hand, the bond length and the bond angle of Cm differ considerably from those of P1 and R3m. Cm has two intermediate bond lengths between short and long bonds: the bond length of 2.935 Ǻ mainly reflects the covalent and ionic bonding structure, while the bond length of 2.976 Ǻ primarily reflects the van der Waals bonding structure. Compared with the bonds in R3m, the short bond in Cm decreases from 2.824 Ǻ to 2.803 Ǻ, and the long bond increases from 3.149 Ǻ to 3.17 Ǻ. This generation of intermediate bonds shows the possibility of the existence of new local minimum points with stronger van der Waals and weaker covalent and ionic bonds; in other words, a weakened Peierls distribution is formed. In particular, Fm3m has a single bond length and angle because the Peierls distortion completely disappears under high-pressure conditions.

**Figure 4:**
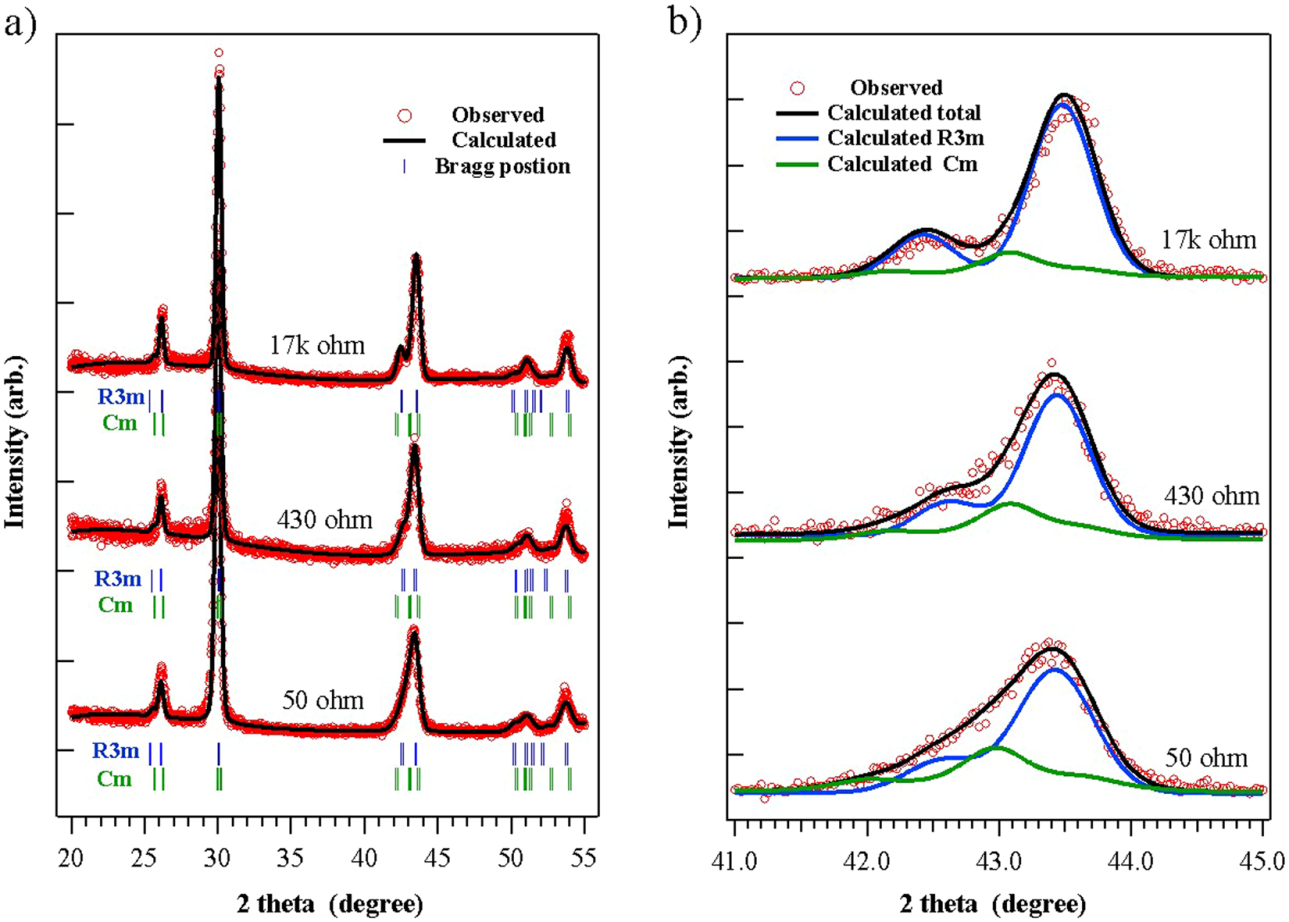
(**a**) Experimentally measured and calculated (Rietveld method) XRD spectra for annealed and laser-irradiated GeTe films. (**b**) Measured and calculated XRD spectra in the range between 40° and 46°. XRD spectra of crystalline samples contain peaks from the R3m (P1), and Cm structures. Samples subjected to laser irradiation had decreased Cm peak intensity and increased resistance.

To confirm the formation of the modeled GeTe crystal structures in real systems, we investigated the crystal structure of the prepared films in detail using the Rietveld refinements method (Fig. 4a). Since it is difficult to distinguish P1 from R3m even in the calculated XRD spectra, both structures were considered to be an R3m structure by Rietveld refinements. The formation of Fm3m was not considered owing to the high Gibbs free energy revealed by the thermodynamic analysis. As shown in Fig. 4, Rietveld refinements reproduce the experimentally observed peak positions, intensity, and shapes associated with R3m and Cm. It is difficult to directly deconvolute the diffraction peak of R3m from that of Cm in the experimental data, owing to peak boarding. Fortunately, the difference between the structures can be clearly identified based on the change in the measured XRD peak shape near 43°, as shown in Fig. 4b. The measured XRD spectrum can be deconvoluted based on the calculated XRD peaks for each structure, in the region from 40° to 46°. Here, P1 and R3m have almost the same spectral shape with two characteristic Bragg diffraction peaks (blue line), whereas Cm exhibits another Bragg diffraction peak between the two Bragg diffraction peaks of R3m and P1. When GeTe was fully crystalized by annealing for 15 min at 250 °C under ambient N2, an asymmetrically broad peak was generated. Thus, the separation of diffraction peaks of R3m and P1 depends on the laser irradiation condition. The resistance increase can be explained here by partial amorphization owing to the laser irradiation^25, 26^. However, since the change in the peak shape is not caused by amorphization, it cannot be directly related to the resistance increase. In particular, the distinctly stronger main peak at ~44° indicates that the crystalline ordering and the grain increase after the laser irradiation^21^. After the laser irradiation, the XRD peaks of the irradiated sample became sharper, while the sample’s resistance increased. Thus, it is more reasonable to assume that the resistance change is related to the crystal structure transformation, not to the amorphization or grain size reduction. Thus, the change in the peak shape of the laser-irradiated sample can be described in terms of a change in the relative presence of R3m (or P1) and Cm. After the laser irradiation, the XRD peaks became sharper, while the sample’s resistance increased. Therefore, it is more reasonable to assume that the resistance change is related to the crystal structure transformation, rather than to the crystalline ordering or grain size increase. Thus, the change in the peak shape of the laser-irradiated sample can be described in terms of a change in the relative presence of R3m (or P1) and Cm. After the laser irradiation, the diffraction peak of Cm becomes weaker, indicating the phase transition from Cm to R3m (or P1). In addition, owing to the small difference between the internal energies of P1 and R3m, these two structures can coexist after the laser irradiation. Therefore, the XRD peaks at 43° and 44.3° can be assigned to both P1 and R3m. The change in the peak shape is well reproduced by changing the composition and stress of R3m and Cm using Rietveld refinements. The lattice parameters calculated using the XRD results in Table 1 show that the lattice parameters (a,b,c) of R3m decrease with decreasing resistance, indicating that compressive stress can be related to the observed resistance increase. As shown in the formation energy diagram of GeTe crystal structures with compressive structures (Fig. 2), Cm can be generated with compressive stress and transformed back to R3m or P1 by reducing the stress. The compressively stressed sample contains more Cm in the XRD results, which is consistent with the thermodynamic analysis results. This strongly supports the existence of Cm structures.

**Figure 5:**
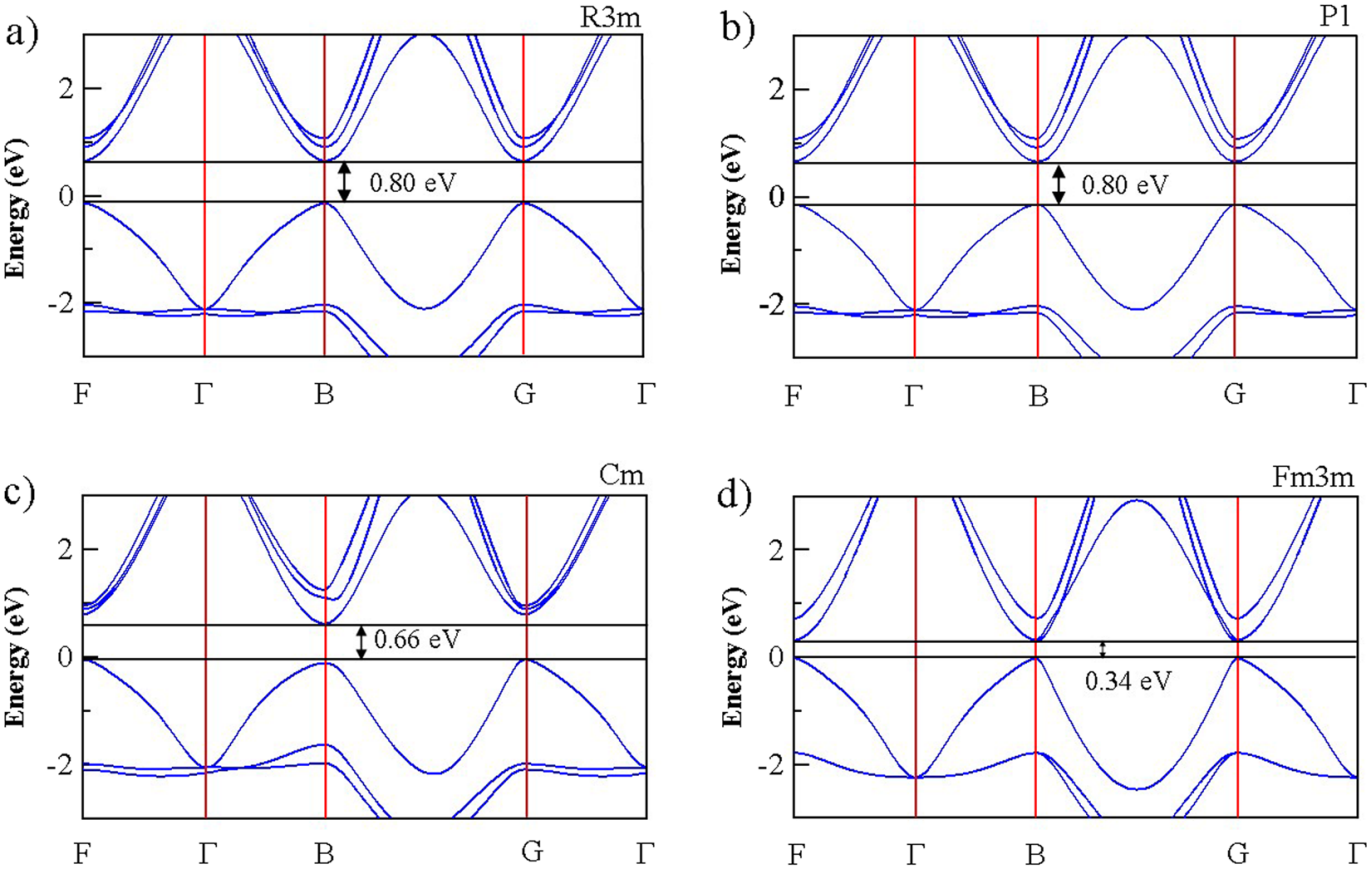
Calculated band structure of each crystalline structure for (**a**) R3m, (**b**) P1, (**c**) Cm, and (**d**) Fm3m. R3m and P1 have almost the same band gap (0.80 eV), while Cm has a lower band gap (0.66 eV) and Fm3m has the lowest band gap (0.34 eV). These various band gaps cause the resistance difference between samples containing different portions of the various crystal structures.

To determine the relationship between the structural transformation and the resistance change, we calculated the band structure for all of the structures, using DFT (Fig. 5). The electronic structures of R3m and P1 were almost the same owing to their similar geometric structures; the band gaps of R3m and P1 were 0.80 eV. A direct band gap in these structures occurred at the B point, similar to the reported band gap of GeTe. The band gap of Cm, ~0.66 eV, was smaller than that of R3m or P1, and the band structure exhibited an indirect band gap from the F point to the B point. However, the valence band maxima were almost the same for F and B. In particular, among the considered GeTe crystalline structures, Fm3m had the smallest direct band gap (0.34 eV) at the B point. These calculation results satisfactorily capture the wide distribution of reported band gaps for crystal GeTe, ranging from 0.3 eV to 0.8 eV, implying that various crystalline structures can be formed during the crystallization of GeTe films. This result suggests that the formation of various crystalline structures with different band structure and resistance can be a fundamental origin of resistance drift in PRAM devices.

**Figure 6:**
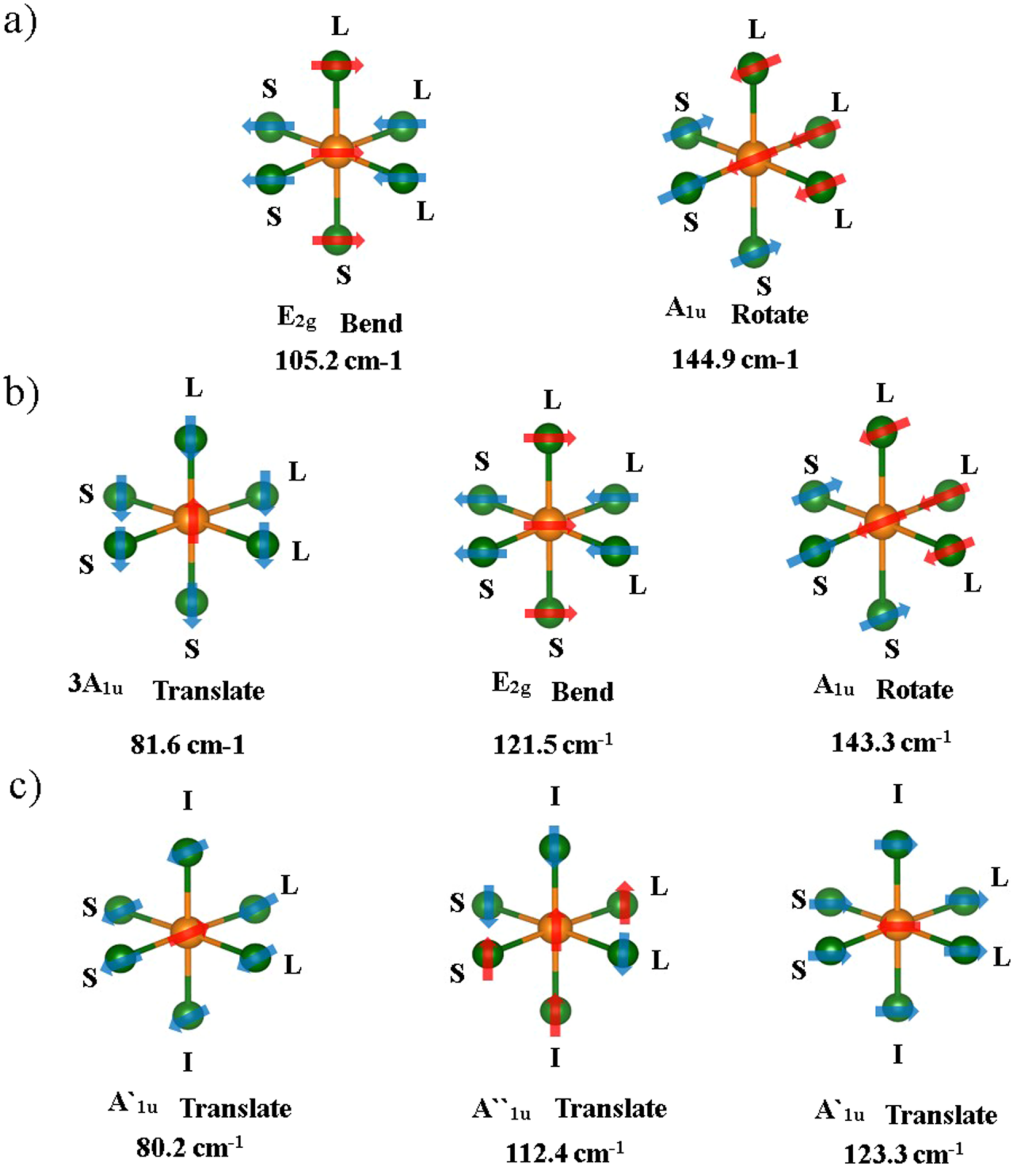
Positions, atomic vibrations, and symmetries of Raman-active modes as predicted by DFT: (**a**) R3m, (**b**) P1, and (**c**) Cm. The Fm3m structure has no Raman-active modes. Movement directions are indicated by arrows and opposite directions are distinguished by red and blue colors. The calculated peak positions and symmetries are shown on the bottom.

Analysis of Raman vibration modes can also provide information on structural distortions. To predict the Raman vibration modes containing atomic structural information, we performed phonon calculations using DFT. The vibration modes and the calculated peak positions are shown in Fig. 6. Interestingly, despite the structural similarity between P1 and R3m, the number of Raman peaks and their positions were very different: the calculated Raman spectrum of P1 featured three peaks at 81.6 cm^−1^, 121.5 cm^−1^, and 143.3 cm^−1^, whereas that of R3m featured only two peaks at 105.2 cm^−1^ and 144.9 cm^−1^. Because the PDOS is similar for P1 and R3m, the dissimilarity reflects their different crystalline symmetries. In P1, a distortion of the bond angle can change the Raman activity of transitional vibration, resulting in very different spectral shapes of P1 and R3m. Therefore, because the Raman vibration can be clearly distinguished even across similar structures, the Raman peak of Cm is also different from those of P1 or R3m. In the case of Cm, the difference in the Raman spectrum is attributed not only to the distinctive symmetry but also to the dissimilar PDOS, as observed in the calculated Raman spectrum of Cm, with three peaks at 80.2 cm^−1^, 112.4 cm^−1^, and 123.3 cm^−1^. Since Fm3m does not exhibit any Raman-active vibrations, it has no Raman mode. The characteristic Raman peaks satisfactorily capture the structural symmetry: i.e., R3m, P1, and Cm had characteristic Raman peaks caused by geometrical symmetry. According to the characteristics table, R3m has one nondegenerate vibration and one doubly degenerate vibration (A + E), whereas P1 has three nondegenerate vibrations (3 A). In addition, Cm has two nondegenerate vibrations along the xz and yz planes, and one nondegenerate vibration along the xy plane (2 A′ + A″). The peak numbers according to the DFT calculations are exactly consistent with the estimated results using the group theory formalism^27–29^. Although other rhombohedral structures of chalcogenide materials with 2:3 stoichiometry feature A_1g_ vibration for the horizontal direction and E_1u_ vibration for the vertical direction relative to the van der Waals layer, R3m of GeTe exhibits a very different behavior. The R3m structure of GeTe has two Raman-active modes, E_2g_ bending vibration and A_1g_ rotation. Unlike the case with chalcogenide hexagonal materials with 2:3 stoichiometry, the Raman-active vibrations of R3m are not parallel or perpendicular to the van der Waals bond layer. Considering P1, although the number of peaks was the same as that expected from the group theory calculations, the vibration modes did not exactly follow the predictions of the group theory calculations. Although P1 is similar to R3m, the Raman peaks of E_2g_ for bending and A_1u_ for rotation arise at different locations owing to the different symmetry of P1. Vibration in the P1 symmetry originates from the triply degenerate (x, y, z) translational vibration. On the other hand, the Raman vibration mode of Cm is very different from those of P1 and R3m, because its chemical structure and structural symmetry are completely different. Therefore, the peak positions in the Raman spectrum of Cm differ from those in the spectra of P1 and R3m. Depending on the symmetry of the Raman vibration, peaks can be attributed to A′_1u_ and A″_1u_ transitional vibration modes.

**Figure 7:**
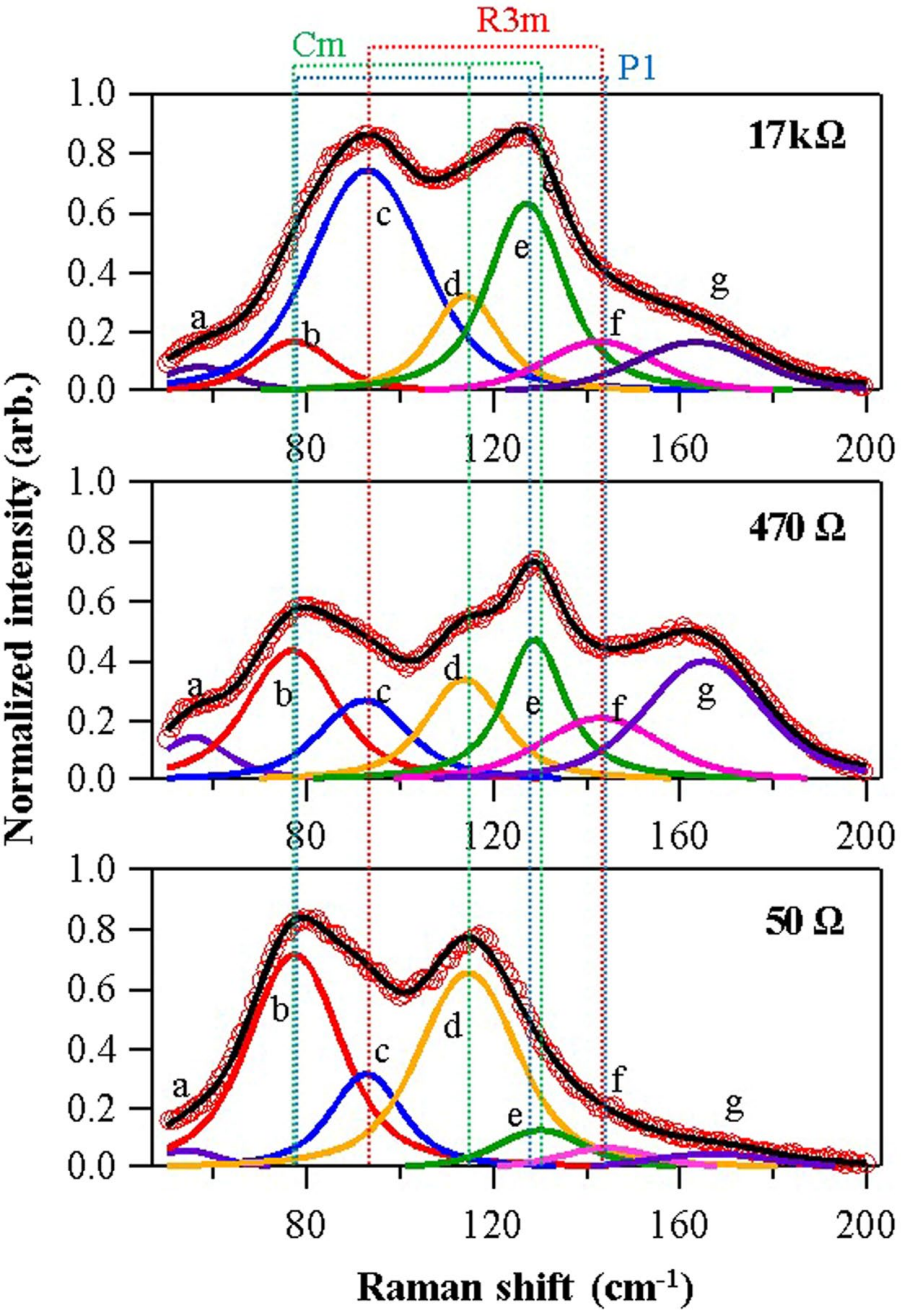
Raman spectra of annealed and laser-irradiated GeTe thin films. Each spectrum can be deconvoluted into seven peaks. Each peak is identified by means of DFT calculations and references. The change in Raman intensity for laser-irradiated samples corresponds to the structural transformations observed in the XRD results.

To verify that the calculated structural characteristics were consistent with those obtained from experimentally acquired Raman spectra, we deconvoluted the Raman spectra and compared the deconvoluted peaks to the calculated vibration characteristics. All Raman spectra of the annealed and laser-irradiated GeTe could be deconvoluted into seven peaks (Fig. 7). In Fig. 7, the peaks are labeled in the alphabetical order. Using the DFT calculations, we confirmed that the peaks b to f were related to the crystal structure of GeTe. The peaks a and g likely originated from defects or from amorphously structured GeTe^11^. Table 2 lists the measured and calculated peak positions as well as their structural origins. Although the relative positions of the Raman peaks reflect the real system well, the peak positions do not directly correspond to the experimental data, owing to the error in the van der Waals force according to the DFT calculations^30^. However, the calculation results allow us to satisfactorily explain the Raman spectra results for laser-irradiated GeTe. Because the second Raman peak of Cm does not overlap with other peaks, the peak positioned at 112.4 cm^−1^ can be used as a measure of the presence of Cm (in experiments, this Raman peak was found to be centered in the 113.9–114.8 cm^−1^ window). We clearly observe that the second peak of Cm decreased with increasing the resistance, which is consistent with the XRD data. Because the Raman peaks of P1 overlap those of R3m and Cm, it is difficult to directly confirm any changes in P1. However, because the second peak of P1 corresponds to the third peak of Cm, changes in P1 can be observed indirectly from changes in the relative intensities of these two peaks. Moreover, the increase in the peak intensity of R3m with increasing resistance is easily observed for the peak in the 92.6–93.0 cm^−1^ window. Shifts in the Raman peak positions are observed for several peaks. The peak shifts observed in the Raman spectra are possibly owing to the internal stress, as shown in the XRD results. However, owing to the overlap and broadening of Raman peaks, which originates from the structures, it is difficult to exactly infer the stress from the Raman peak shifts.

**Table 2:** Raman peak positions, origins, and symmetries.

Peak notation	Experimental Raman shift (cm^−1^)	Calculated Raman shift (cm^−1^)	Structure	Space number	Symmetry
c	92.6–93.0	105.2	R3m	160	E_2g_
f	142.7–145	144.9			A_1u_
b	77.0–77.6	81.6	P1	1	3A_1u_
e	127.4–129.7	121.5			E_2g_
f	143.1–145.0	143.3			A_1u_
b	77.5–79.7	80.2	Cm	8	A′_1u_
d	113.9–114.8	112.4			A″_1u_
e	127.3–130.3	123.3			A′_1u_

Although the peaks at 58 cm^−1^ and 163 cm^−1^ cannot be identified in our calculation results, according to a previously reported result these peaks arise from amorphous GeTe. The intensity of the peak at 163 cm^−1^ caused by amorphous GeTe is not consistent with the following resistance change; this peak has its highest intensity for the sample with the resistance of 430 Ω, not for the sample with the highest resistance of 17 kΩ. This provides additional evidence in support of our conclusions that the resistance change in the present experiments did not arise only from the amorphous structure of the amorphized GeTe.

**Figure 8:**
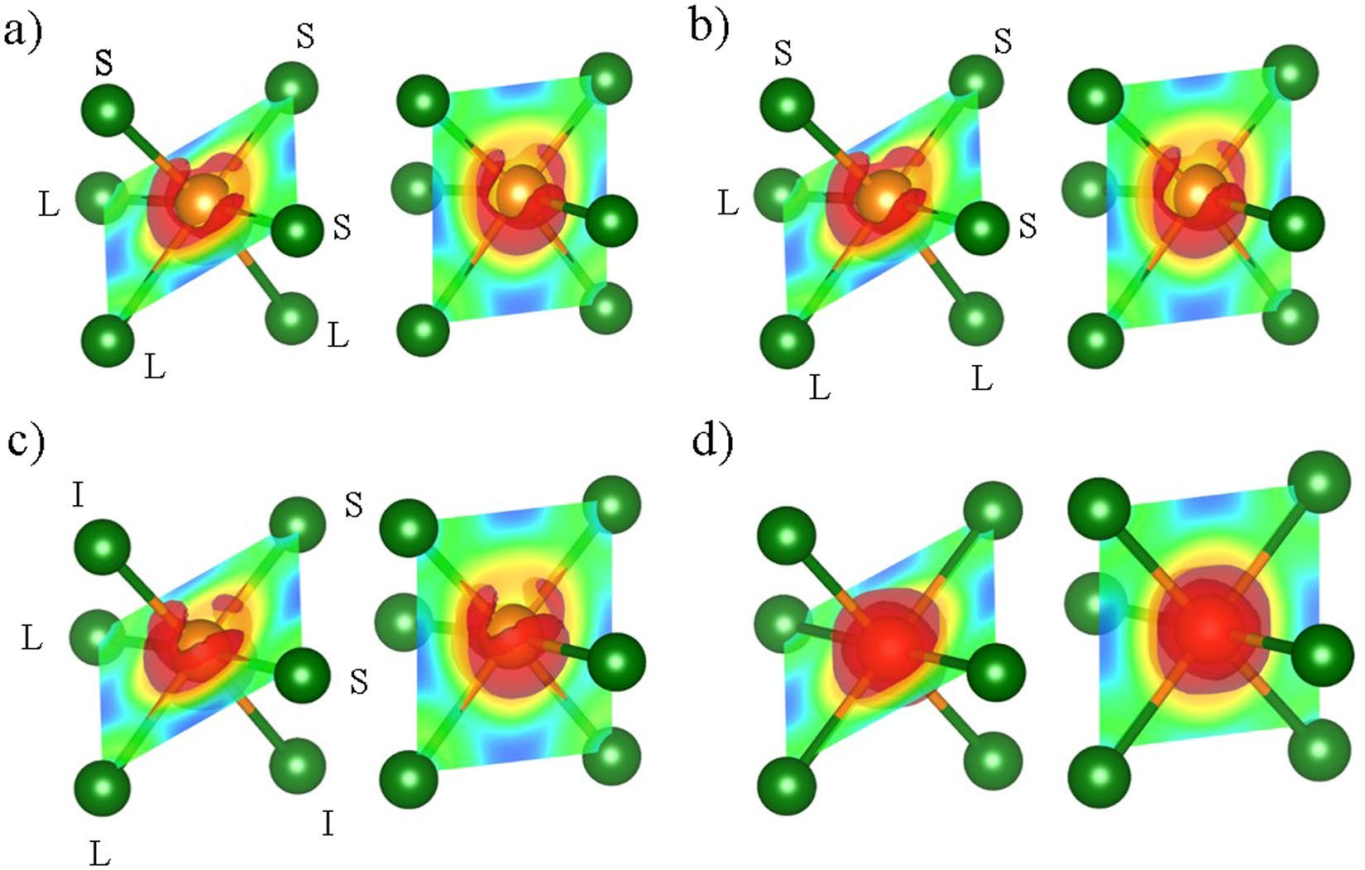
Electron localization functions of the different structures, as determined by the DFT calculations: (**a**) R3m, (**b**) P1, (**c**) Cm, and (**d**) Fm3m. Red isosurfaces correspond to ELF = 0.85. Left and right figures for each structure exhibit different 2D planes that contain bonds. In the 2D cross-section view, the regions of high ELF and low ELF are represented with red color and blue color, respectively.

To analyze the bonding properties of the different structures, especially the intermediate bonding of Cm, electron localization functions (ELFs) were calculated by DFT^31^. The ELF is directly related to the electron distribution probability in the range bounded by the minimal ELF (0) and maximal ELF (1). The isosurface of 0.85 ELF and a two-dimensional (2D) cross-section with bonding planes are shown in Fig. 8. R3m and P1 show isosurfaces of similar morphology. If the bond is close in character to a covalent bond, the ELF increases in the bonding direction. For R3m and P1, localized high-ELF points are observed for three short bonds. In the opposite direction corresponding to long bonds, a broad shell is observed, which is caused by a lone pair and van der Waals bonds. The Cm structure exhibits more interesting properties. In particular, an intermediate bond caused by both the covalent bond and the van der Waals bond is observed, although two short bonds in Cm are similar to those in R3m and P1. With the bond length difference of only 0.015 Å, the shorter bond becomes more like a covalent bond and the longer bond becomes more like a van der Waals bond. The formation of the Cm structures indicates that one or several minimum energy points can be generated by the bond length changes of van der Waals and covalent bonds, namely the strengthening of the van der Waals bond and weakening of the covalent bond. Based on this structural change, various stable bonding lengths in GeTe can be explained. On the other hand, the Fm3m structure is very different from the other three structures. Because Fm3m has six identical covalent bonds, there is a symmetric pop up isosurface in the bonding direction. The distorted structure in GeTe can be released by means of a stabilization process to control the repulsive energy of the Peierls distortion. Therefore, various crystalline structures can be generated by changing the van der Waals and covalent bonds. In reality, distorted structures are induced during the phase transition process, owing to the volume changes during this transformation between amorphous and crystalline structures. We successfully confirmed the generation of various structures with different physical characteristics, such as different bond types and band structures.

## Conclusions

In this paper, we investigated possible crystal structures of GeTe using DFT calculations. We noted that four crystal structures can be generated: R3m, P1, Cm, and Fm3m. Among these, P1 and Cm have been considered for the first time in the present study. By investigating the relationship between volume and internal energy, we verified that P1, R3m, and Cm can coexist in crystalline GeTe. In addition, based on the thermodynamic analysis, we predict that the preferred phase can be easily changed in the PRAM switching process accompanying the modulation of stress and temperature. By analyzing the XRD spectra of crystalline GeTe, we verified that Cm and P1 or R3m coexist in the real system and the relative presence of different structures can be modulated by compressive stress. Moreover, we show that Cm transforms to R3m or P1 with stress relaxation by laser irradiation. By means of this crystal structure transformation, resistance is increased owing to the higher band gap of P1 or R3m compared with that of Cm. Finally, we confirmed that the Raman spectra of annealed samples are well consistent with the vibrational modes from the new structure obtained by calculations. The presence of many peaks in the Raman spectra of crystalized GeTe can be explained by the coexistence of various structures. The structural transformation of Cm into R3m or P1 is also consistent with the observed changes in resistance. In addition, we suggest that the resistance change in GeTe can be modulated by generating an intermediate bonding state. Generation of these crystal structures can explain the observed resistance change, resulting in the resistance drift during the phase transition.

The band gap, bonding length, and vibration modes of Cm are distinct, compared with those of R3m and P1. These unique properties of Cm originate from intermediate bonding between layers, which is not fully covalent and not fully van der Waals. The existence of Cm implies that one or several minimum energy points can be generated through the bond length changes arising from the strengthening of the van der Waals bonds and weakening of covalent bonds. This intermediate bonding is one possible explanation for a variety of stable bonding lengths observed in amorphous structures, as well as the easy phase transition. Moreover, the formation of intermediate bonds can cause structural instability, which can directly affect device reliability.
